# The proline-rich peptide Bac7(1-35) reduces mortality from *Salmonella typhimurium *in a mouse model of infection

**DOI:** 10.1186/1471-2180-10-178

**Published:** 2010-06-23

**Authors:** Monica Benincasa, Chiara Pelillo, Sonia Zorzet, Chiara Garrovo, Stefania Biffi, Renato Gennaro, Marco Scocchi

**Affiliations:** 1 Department of Life Sciences, University of Trieste, Via Giorgieri 1, 34127 Trieste, Italy; 2 Optical Imaging Laboratory, CBM, Area Science Park, Trieste, Italy

## Abstract

**Background:**

Bac7 is a proline-rich peptide with a potent *in vitro *antimicrobial activity against Gram-negative bacteria. Here we investigated its activity in biological fluids and *in vivo *using a mouse model of *S. typhimurium *infection.

**Results:**

The efficacy of the active 1-35 fragment of Bac7 was assayed in serum and plasma, and its stability in biological fluids analyzed by Western blot and mass spectrometry. The ability of the peptide to protect mice against *Salmonella *was assayed in a typhoid fever model of infection by determination of survival rates and bacterial load in liver and spleen of infected animals. In addition, the peptide's biodistribution was evaluated by using time-domain optical imaging. Bac7(1-35) retained a substantial *in vivo *activity showing a very low toxicity. The peptide increased significantly the number of survivors and the mean survival times of treated mice reducing the bacterial load in their organs despite its rapid clearance.

**Conclusions:**

Our results provide a first indication for a potential development of Bac7-based drugs in the treatment of salmonellosis and, eventually, other Gram-negative infections. The *in vivo *activity for this peptide might be substantially enhanced by decreasing its excretion rate or modifying the treatment schedule.

## Background

The spread of multi-resistant bacterial pathogens poses a serious threat to the global society in light of commonly appearing hospital- and community-acquired drug-resistant infections. It is therefore urgent to search for new potent antimicrobial agents coping with arising pathogen invasion and, at the same time, minimising the probability of resistance induction in bacteria.

Antimicrobial peptides (AMPs) are widely recognized as promising alternatives to the currently used antibiotics and fungicides [1,2]. AMPs are widespread in living organisms and constitute an important component of innate immunity to microbial infections [3]. In mammals, they are produced by granulocytes, macrophages and most epithelial cells [4,5]. Amino-acid sequences of the vast majority of AMPs share cationic and amphipathic properties that allow their insertion into lipid bilayers and can lead to alteration of biological membrane functions [6]. Initial characterization studies linked these properties to antimicrobial killing activity. However, further data indicated that this is not the only mode of action and that more subtle mechanisms might mediate the interaction with, and effect on target microbes, as well as the specificity and toxicity of peptides.

The proline-rich peptides (PRPs) are a distinctive group of AMPs, isolated in different animal sources, which display an unusually high content of proline and arginine residues in their sequences [7,8]. These peptides show a spectrum of activity limited to Gram-negative bacteria and appear to have a stereospecific mode of action mediated by the internalization of the peptides into the cytoplasm without extensive membrane damaging effects [7].

Bac7 is a linear, 60-residue proline-rich peptide of bovine origin corresponding to the C-terminal antimicrobial domain of a specific protein precursor of cathelicidin family [9]. Previous studies demonstrated that Bac7, and its C-terminal truncated form Bac7(1-35), have a potent *in vitro *activity against many Gram-negative bacteria including *Enterobacteriaceae*, particularly *Salmonella *spp., and the genera *Pseudomonas, Acinetobacter*, and *Sinorhizobium *[10-12], while it is inactive against most of the Gram-positive bacteria. Bac7(1-35) is also active against multi-resistant clinical isolates [10] and is able to neutralize endotoxin in experimental rat models of Gram-negative septic shock [13]. In contrast to most AMPs, this peptide is not toxic to mammalian cells at concentrations well above those effective against microbes [13,14]. In this respect, Bac7(1-35) is internalized into eukaryotic cells through a pinocytic process [14,15], but enters bacterial cells through a mechanism mediated by the membrane protein SbmA/BacA [12,16]. These features suggest that Bac7 and its fragments might be used *in vivo *without being toxic to the host and be effective also against intracellular pathogens.

Despite the high potential of many AMPs as antimicrobial agents [17], in most cases, their residual toxicity towards host cells and their rapid degradation and/or inhibition by components of biological fluids represent a real obstacle to their development as therapeutic molecules [18,19].

In this study we investigated the *in vitro *activity of Bac7(1-35) in a more physiological context, such as in murine serum and plasma, and the *in vivo *potential in a murine infection model of typhoid fever. Results indicate that the peptide remains substantially active at the site of infection and reduces significantly the mortality of infected animals despite its rapid clearance.

## Results and Discussion

### Antibacterial activity of Bac7(1-35) in serum or plasma

**Figure 1:**
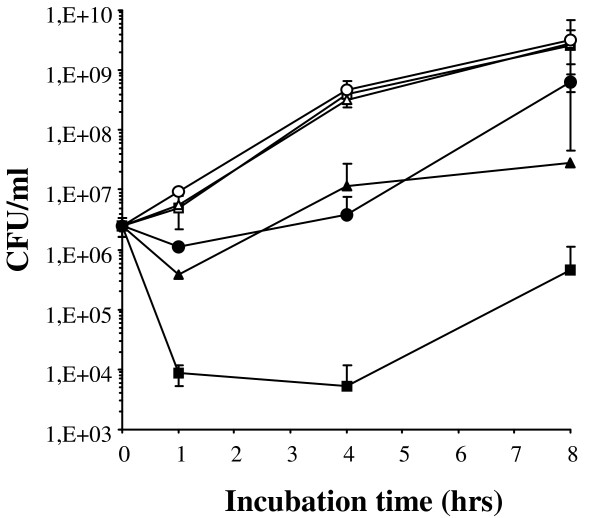
**Antimicrobial activity of Bac7(1-35) in the presence of biological fluids**. Kinetics of the bactericidal activity of 10 μM Bac7(1-35) against *S. enterica *ATCC 14028 in the absence (filled squares) or in the presence of 66% murine serum (filled circles), or 66% murine plasma (filled triangles). Bacterial growth without peptide is indicated by empty symbols. Results represent the mean ± SD of three independent determinations performed in triplicate.

Previous results showed that Bac7(1-35) has a potent *in vitro *activity against Gram-negative bacteria [10]. Before testing whether this peptide can also be active *in vivo*, we assayed its antibacterial activity *in vitro *in the presence of body fluid components. When killing kinetics assays were performed in the presence of 66% murine plasma or serum, the activity of Bac7(1-35) towards *Salmonella enterica *serovar Typhimurium was reduced although still detectable (Figure 1). In particular, after 1h-incubation with serum or plasma, Bac7(1-35) (10 μM) reduced the number of CFU by 0.5-1 log *vs *2.5 log detected in the absence of these biological fluids. To obtain a comparable reduction of CFU without the presence of plasma/serum, 1 μM peptide should be used. At longer incubation times, the activity of the proline-rich peptide seemed further inhibited, especially by murine serum (Figure 1). We also assayed the effects of serum albumin, the most abundant protein in blood, on the peptide activity. In contrast to what observed for other AMPs [19], the bactericidal activity of Bac7(1-35) did not change upon addition of 40 mg/mL BSA, a concentration corresponding to that present in the blood (data not shown).

### Stability of Bac7(1-35) in serum and plasma

**Figure 2:**
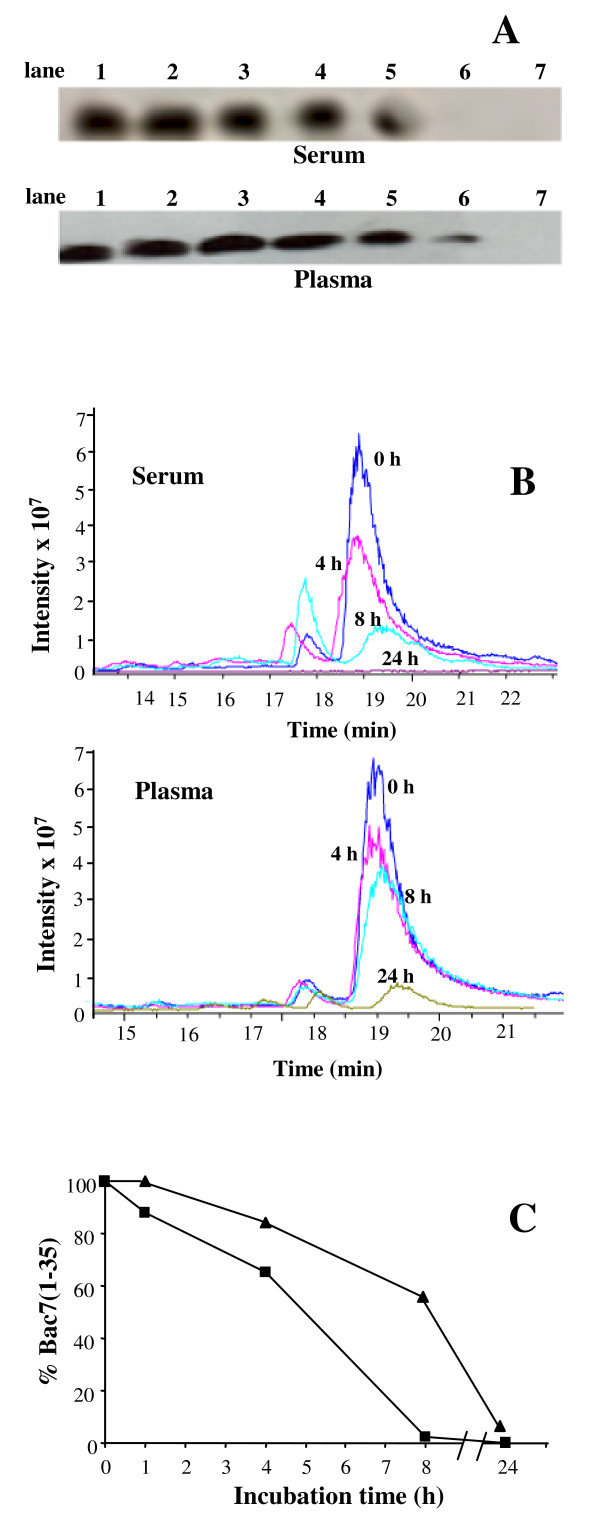
**Bac7(1-35) stability in blood fractions**. (A) Western blot analysis of Bac7(1-35) incubated for different times at 37°C in 25% murine serum or plasma. Lane 1: 0.5 μg Bac7(1-35); lanes 2-6: Bac 7(1-35) after incubation with murine serum or plasma for respectively 0, 1, 4, 8, 24 hrs; lane 7: serum or plasma alone. (B) MC-LC chromatograms of Bac7(1-35) incubated at 37°C in 25% murine serum or plasma. (**C**) The percentages of Bac7(1-35) with respect to the t_0 _control were calculated following LC-MS analysis (see section Methods for further details) after incubation of the peptide with murine serum (filled squares) or plasma (filled triangles) for different times.

Inhibition of the peptide due to enzymatic degradation by blood proteases was taken into account to explain the reduced activity of Bac7(1-35) in biological fluids. The stability of Bac7(1-35) was therefore evaluated by incubating the peptide up to 24 h with murine plasma or serum followed by Western blot analysis. Immunodetection indicated a slow and progressive reduction of the band corresponding to intact Bac7(1-35), which disappeared after 24 h-incubation in serum (Figure 2A). The degradation of Bac7(1-35) in plasma was slower (Figure 2A), suggesting that the activation of proteases of the coagulation cascade in serum may contribute to the faster peptide degradation in this medium. LC-MS analysis indicated that the amount of intact Bac7(1-35) in murine serum decreases by 10% after 1 h of incubation and that the peptide was almost completely degraded after 8 h (Figure 2B and 2C). The degradation process is slower in plasma than in serum, (Figure 2B and 2C), confirming the result observed in the Western blot analysis, while in PBS alone, no peptide degradation was observed even after several days of incubation at 37°C.

No fragments of Bac7(1-35) were detected by LC-MS analysis. This may due to generation of hydrophilic fragments of a few residues by responsible protease(s), which are not detectable by either anti-Bac7 antibodies or LC-MS. Enzymes such as trypsin-like serine proteases, which may cleave at the many Arg residues present in the sequence of Bac7(1-35), might have this effect. However, these results clearly indicate that the peptide should be quite stable in blood and its degradation occurs only after several hours, suggesting that the decreased activity of Bac7(1-35) is only in part due to its degradation.

### *In vivo *toxicity

As a first step to evaluate the therapeutic potential of Bac7(1-35), its *in vivo *toxicity was determined in Balb/c and CBA/Ca mice after injection via i.p. of increasing single peptide doses. No apparent toxic effect was observed when the peptide was administered i.p. up to 75 mg/kg, but the mice receiving the highest peptide dose (150 mg/kg) died 3 days post injection. This result confirms that Bac7(1-35) is much less toxic than other cathelicidin-derived peptides such as those belonging to the α-helical group [20] and, in this respect, it behaves similarly to insect proline-rich AMPs. For example, pyrrhocoricin protected mice against *E. coli *infection, and showed no toxicity up to the maximal applied dose i.v. of 50 mg/kg. Drosocin is completely devoid of toxicity to healthy animals when used via i.v. at 100 mg/kg [8]. On the contrary, lytic peptides such as BMAP-27 and -28 are toxic via i.p. already at 10-15 mg/kg [20].

### *In vivo *Bac7(1-35) activity in a mouse model of typhoid fever

**Figure 3:**
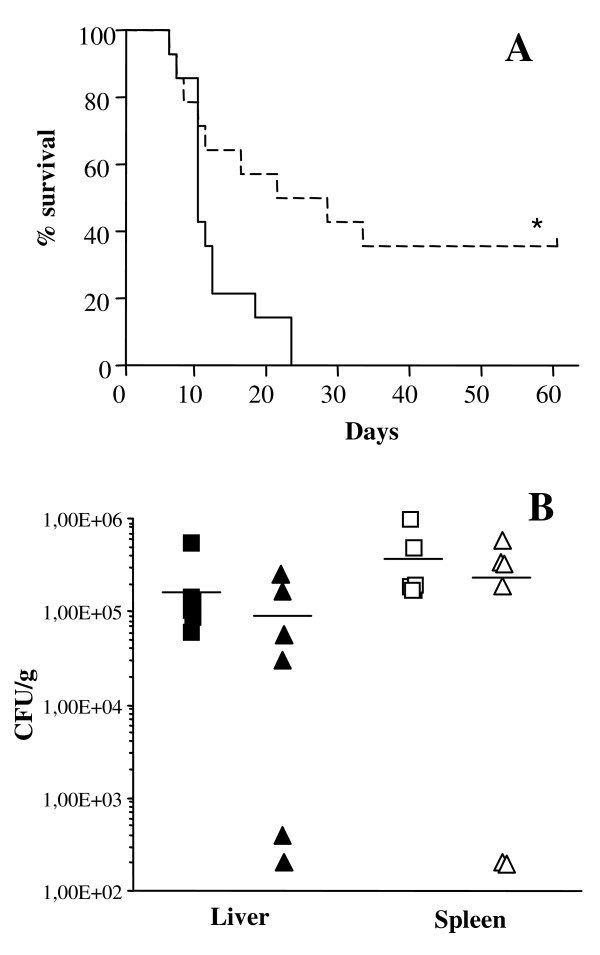
***In vivo *activity of Bac7(1-35)**. Survival curves (**A**) and viable bacterial counts in liver and spleen homogenates (**B**) of mice infected with *S. enterica *after treatment via i.p. with Bac7(1-35) are shown. CBA/Ca mice were infected via i.p. with *S. enterica *ATCC 14028 (10^2 ^CFU/mouse) and Bac7(1-35) at 30 mg/kg was immediately injected via i.p. after bacterial challenge (dotted line). Control mice were given 0.2 ml of PBS (continuous line). Mice were monitored for survival over a 60-day period after infection. *p < 0.05 treated *vs *untreated mice. Three days after bacterial infection, untreated (squares) and peptide-treated (triangles) mice were killed, and liver (full symbols) and spleen (empty symbols) homogenates were prepared as described in section Methods. Results are expressed as number of CFU/g organ; bars represent the mean value for each group.

The potential of Bac7(1-35) to protect mice from a bacterial challenge was tested by a mouse model of *Salmonella *infection. Infected mice develop a systemic disease characterized by rapid bacterial multiplication in the liver and spleen that resembles typhoid fever caused by *Salmonella *serovar Typhi in humans [21]. Cell-mediated immunity and macrophage activity play a key role in defence against murine salmonellosis, and it has been shown that these immune responses are lacking in Balb/c mice [22,23] so that also the antibiotic ciprofloxacin failed to prevent fatal *S. typhimurium *disease in this mouse strain [22]. For this reason, we preferred to use CBA/Ca mice that show a lower susceptibility to *Salmonella *infection [22] to study the antimicrobial properties of Bac7(1-35). Nevertheless, an acute infection may be induced by i.p. injection of less than a hundred of CFU/mouse. Male CBA/Ca mice were infected via i.p. with a lethal dose of *S. typhimurium *ATCC 14028 (1 × 10^2 ^CFU/mouse), followed by i.p. injection of peptide at 30 mg/kg. The number of survivors was monitored for 60 days and compared to that of control mice that only received the lethal bacterial challenge. The survival curves of untreated and peptide-treated mice are significantly different (p = 0.01); the mean survival time of control mice was 10 days, while the treatment of infected mice with Bac7(1-35) increased the mean survival time to 24.5 days. It is worth noting that 36% of the infected mice treated with Bac7(1-35) were completely cured with respect to 0% survival for untreated animals (p = 0.04) (Figure 3A).

In parallel to survival experiments, a group of mice was also analyzed for bacterial load at 3 days post-inoculation, when the infected animals did not show any visible sign of disease. Viable bacterial cells were counted in murine liver and spleen of infected mice and results are reported in Figure 3B. The number of viable bacterial cells in liver and spleen homogenates decreased significantly in the animals treated with the peptide at 30 mg/kg, despite a remarkable variability in each group. In 1/3 of the animals bacteria were undetectable in both the spleen and liver. This result is in keeping with the percentage of mice cured extrapolated by the survival curve (Figure 3A).

Given that i.p. injection of as few as 100 salmonellae is lethal for mice, the increased survival times and the eradication of the infection in 1/3 of the peptide-treated animals is a promising result. In addition, the protective role showed by Bac7(1-35) suggests that the peptide may exert its bactericidal action also in infected cells, since *S. typhimurium *is an intracellular pathogen and Bac7(1-35) is able to penetrate host cells [14,15].

### *In vivo *Time-Domain Optical Imaging

Following the results with the mouse model of infection, we investigated the *in vivo *biodistribution of Bac7(1-35) by using a time-domain optical imaging instrument [24] and a derivative of Bac7(1-35), fluorescently labelled with the dye Alexa680, showing an antimicrobial activity comparable to that of the unlabelled peptide (data not shown).

**Figure 4:**
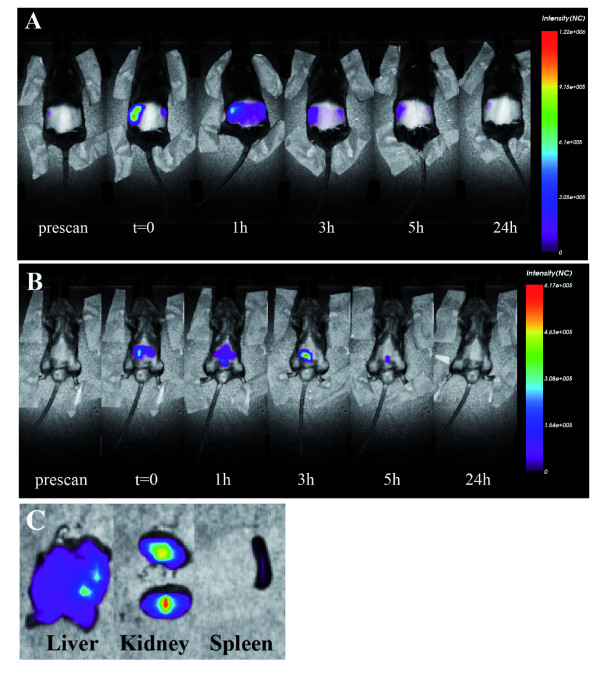
**Biodistribution of Bac7(1-35)-Alexa680 in healthy mice after i.p. injection**. (**A**) The animal was placed in prone position, fluorescence emission in regions of interest encompassing the kidneys were acquired at indicated times post-injection and normalized. (**B**) The animal was placed in supine position, fluorescence emission in regions of interest encompassing the thorax and abdomen was acquired at indicated times post-injection and normalized. (**C**) *Ex vivo *images of organs at 5 hours after i.p. injection. Imaging of the organs was performed immediately after sacrifice: laser power and integration time were optimized while keeping constant scan step to compare fluorescence intensities after normalization. The images are representative of two independent experiments with comparable results.

The Bac7(1-35)-Alexa680 peptide shows a fast elimination kinetics after i.p. injection, characterized by a specific fluorescence intensity signal in the kidney first and then in the bladder. The compound reaches the kidney and the bladder in respectively 1 and 3 hours after the injection. The *in vivo *and *ex vivo *analyses performed after 24 h confirm that the compound has been totally excreted (Figure 4). These results suggest that the labelled compound does not accumulate in any particular organ except those involved in the elimination processes, kidney and bladder, which result completely free of Bac7(1-35)-Alexa680 one day after injection.

It is well known that mice eliminate drugs thought kidney much more quickly than humans [25]. As no nefrotoxic compounds causing renal dysfunction were used to alter pharmacokinetic parameters [25], the very rapid clearance of the peptide may likely have limited its activity against pathogens after injection in the animals. In the light of this observation, the antibiotic activity of Bac7(1-35) may be improved in the future by slowing the kinetics of its renal excretion.

## Conclusions

In conclusion, with this study we have shown that Bac7(1-35) may exert antibacterial activity also *in vivo*, in a mouse model of infection resembling typhoid fever in humans. This model is particularly challenging in mice due to the extremely low lethal dose of *S. typhimurium*. Intraperitoneal injection of Bac7(1-35) at 30 mg/Kg increased significantly the survival rate of infected mice and the mean survival times suggesting that it inactivates most of the inoculated bacteria in spite of a partial inhibition due to unknown blood components and a very *fast renal excretion rate*.

In the light of these observations, the results here reported provide encouraging evidence for a future development of a Bac7-based drug in the treatment of Gram-negative infections. Its *in vivo *efficacy might be improved by decreasing its clearance rate, for instance by conjugation of the peptide with a drug delivery system. Moreover, its effectiveness can also be improved by changing the treatment regimen, for example with repeated dosing. These studies are currently in progress.

## Methods

### Peptide synthesis and labelling

The N-terminal fragment 1-35 of Bac7 was synthesized, purified and stored as described [11]. Bac7(1-35) was fluorescently-labelled via linkage of the thiol-reactive dye ALEXA FLUOR^® ^680 C_2_-maleimide (Invitrogen, Carlsbad, CA) to a specifically added C-terminal cysteine residue. Briefly, the fluorophore ALEXA FLUOR^® ^680 (1 mg) was dissolved in 100 μL DMSO, and added drop wise to 30 mL Na-phosphate buffer 10 mM, pH 7, under nitrogen bubbling in the dark. Four mg of Bac7(1-35)-Cys were dissolved in this solution; after 1 h incubation at room temperature with stirring, a new aliquot of peptide (4 mg) was added and the solution was left overnight at 4°C. At the end of the incubation time, an excess of cysteine (10 mg) was added in this solution to scavenge the excess of thiol-reactive reagent. The solution was left with stirring for 1-2 h and the labelled peptide was purified by RP-HPLC.

### Antibacterial activity in serum and plasma

Murine plasma obtained using 2% (v/v) Na-citrate as an anticoagulant, and serum were prepared and stored at -20°C until use. The bactericidal activity of Bac7(1-35) against *Salmonella enterica *serovar Typhimurium ATCC 14028 was determined by a killing kinetics assay [11]. Mid-logarithmic phase *S. enterica *cultures were diluted in murine serum or plasma (66% v/v final concentration) or BSA (40 mg/mL) (Sigma) to give approximately 1 × 10^6 ^cells/ml, and incubated with 10 μM Bac7(1-35) in a shaking water bath at 37°C for different times. Samples were withdrawn, diluted and plated to allow colony counts [11].

### Peptide stability in biological fluids

To test the peptide stability in biological fluids, 120 μg of Bac7(1-35) were incubated in 200 μL of PBS containing 25% (v/v) murine serum or plasma at 37°C, or in PBS alone. At different times, aliquots of samples were diluted 1:5 in sample buffer (12% SDS, 6% dithiothreitol, 40% glycerol, 0.05% bromophenol blue, 150 mM Tris-HCl, pH 7), incubated for 15 min at 60°C and analyzed on a 16% Tricine/SDS gel. Proteins were then blotted onto nitrocellulose membrane (Whatman), and incubated overnight with shaking at 4°C in 40 mM Tris-HCl, pH 7.5, 5% non-fat milk, 0.05% Tween 20, 200 mM NaCl (blocking solution). Samples were incubated for 90 min with 1:1000 rabbit anti-Bac7(1-35) IgG, diluted in blocking solution, followed by a HRP-conjugated anti-rabbit IgG (Sigma-Aldrich). The ECL detection system (GE Healthcare) was used to develop the Western blots.

### LC-MS analysis

Bac7(1-35) peptide (50 μg) was incubated in 250 μL of PBS containing 25% (v/v) of murine serum or plasma at 37°C. At different time intervals (0, 1, 2, 4, 8 and 24 h), aliquots of 25 μL (corresponding to 5 μg of peptide) were added to 65 μL of cold 0.5% (v/v) TFA in H_2_O, kept on ice for 5 min and than centrifuged at 10.000 × g for 5 min. The LC-MS analysis of supernatants were carried out as described [26], using a standard curve to calculate the peptide concentration.

### Animals

Male Balb/c and CBA/Ca mice of approximately 20 g and 6 weeks of age were obtained from Harlan Laboratories (Udine, Italy) and maintained under pathogen-free conditions. All the experimental procedures were performed according to the guidelines of the European (86/609/EEC) and the Italian (D.L.116/92 and subsequent addenda) laws and approved by the Italian Ministry of University and Research as well as by the Animal Experimentation Committee of the University Animal House.

### *In vivo *studies

The *in vivo *toxicity of Bac7(1-35) was investigated by injecting mice via i.p. with increasing amounts of the peptide dissolved in apyrogen PBS (0.2 ml per mouse). The controls received the vehicle alone. Animal behavior and survival were monitored over a 14-day period.

Inocula containing 10^2 ^CFU/mouse of *S. enterica *ATCC 14028, expected to result in 90-100% mortality in 4-6 days for Balb/c [27] and 10-18 days for CBA/Ca, were prepared by diluting log-phase bacterial cultures in sterile PBS. Ten mice were infected intraperitoneally and monitored for survival over a 60-day period after infection. Test peptide (30 mg/kg) was injected via i.p. after bacterial challenge. The choice of dose was based on preliminary data obtained with lower doses (data not shown) and on results reported in the literature for structurally similar peptides. Control mice were given 0.2 ml of PBS. The experiment was repeated two times and comparable results were obtained. The analysis of survival curves was conducted using the Kaplan-Meyer method and successive statistical evaluation by the Logrank test. Significance of percentage differences among groups was assessed by using the Fisher exact test. Values of p < 0.05 were considered statistically significant.

### Viable colony counts in murine liver and spleen homogenates

Three days after bacterial infection, a group of 3 untreated and 3 peptide-treated mice were killed by cervical dislocation, and liver and spleen were removed. The organs were weighed, homogenized separately and dissolved in PBS. Suitable dilutions of 50 μL of the homogenate in PBS were plated in duplicate on Mueller-Hinton agar (Difco). The plates were then incubated at 37°C overnight to allow colony counts. Results are expressed as number of CFU/g of organ. This assay was repeated two fold.

### Mice preparation and treatment for *in vivo *Time-Domain Optical Imaging

The day before the treatment, healthy CBA mice were anesthetized by an intramuscular injection of a diluted mixture (1:5 in PBS) composed by 0.4 mL Zoletil 100 and 0.25 mL Rompun 2% (3 μL/g body weight), and shaved in the regions of interest to avoid laser scattering caused by hair. The following day, mice were anesthetized using a gaseous anaesthesia system (2Biological Instruments, Italy), based on isoflurane mixed to oxygen and nitrogen protoxide. Anaesthesia was first induced with 2% isoflurane in a pre-anaesthesia chamber and then the animals were placed inside the eXplore Optix in the presence of 1% isoflurane. Two mice were then injected intraperitoneally with 36.6 μg/mouse of Bac7(1-35)-Alexa680, corresponding to 6.9 nmol ALEXA FLUOR^® ^680, one monitored in the abdominal region and the other in the renal region for 24 hours. A blank image was acquired before treatment of each animal and this was subtracted to the images of the treated animal. Experiment was repeated two times.

The small-animal time-domain eXplore Optix preclinical imager (GE Healthcare) was used in this study. In all imaging experiments, a 670 nm pulsed laser diode with a repetition frequency of 80 MHz and a time resolution of 12 ps light pulse was used for excitation. The fluorescence emission at 700 nm was collected and detected through a fast photomultiplier tube and a highly sensitive time-correlated single-photon counting system. Two-dimensional scanning regions of interest (ROI) were selected and the laser power, integration time and scan step were optimized according to the signal emitted. The data were recorded as temporal point-spread functions, and the images were reconstructed as fluorescence intensity and lifetime.

## Authors' contributions

MB performed antimicrobial assays, *in vivo *studies, and contributed to write the manuscript. CP performed peptide' stability experiments, antimicrobial assays and helped to draft the manuscript. SZ participated in the design of the *in vivo *study and analysis of its results. CG and SB participated in biodistribution studies with *in vivo *Optical Imaging and analysis of the results. RG participated in study design and coordination and helped to edit the manuscript. MS conceived of the study, drafted and wrote the manuscript. All authors have read and approved the final manuscript.
